# Forward Signaling by Unipolar Brush Cells in the Mouse Cerebellum

**DOI:** 10.1007/s12311-015-0693-5

**Published:** 2015-07-02

**Authors:** Stijn van Dorp, Chris I. De Zeeuw

**Affiliations:** Netherlands Institute for Neuroscience, Meibergdreef 47, 1105 BA Amsterdam, The Netherlands; Department of Neuroscience, Erasmus Medical Center, P.O. Box 2040, NL-3000 CA Rotterdam, The Netherlands

**Keywords:** Electrophysiology, Patch clamp, Vestibulocerebellum, Synaptic transmission

## Abstract

Unipolar brush cells (UBCs) are glutamatergic interneurons prominently present in the granular layer of the vestibulocerebellum. UBCs engage in extensive synaptic contact with a single presynaptic mossy fiber and signal to downstream granule cells through an elaborate network of mossy fiber-like axons. Ultrastructural examinations and electrophysiological recordings in organotypic slice cultures have indicated that UBCs target not only granule cells but also other UBCs, thus forming chains of two or perhaps more interconnected UBCs. In this report, we show recordings of spontaneous and evoked (di)synaptic events in granule cells and UBCs in fresh cerebellar slices from juvenile mice (5–7 weeks). The patterns of arrival of synaptic events were consistent with the presence of a presynaptic UBC, and recordings from UBCs displayed spontaneous protracted synaptic events characteristic of UBC excitatory synaptic transmission. These results highlight that chains of UBCs could further extend the temporal range of delayed and protracted signaling in the cerebellar cortical network.

## Introduction

**Fig. 1:**
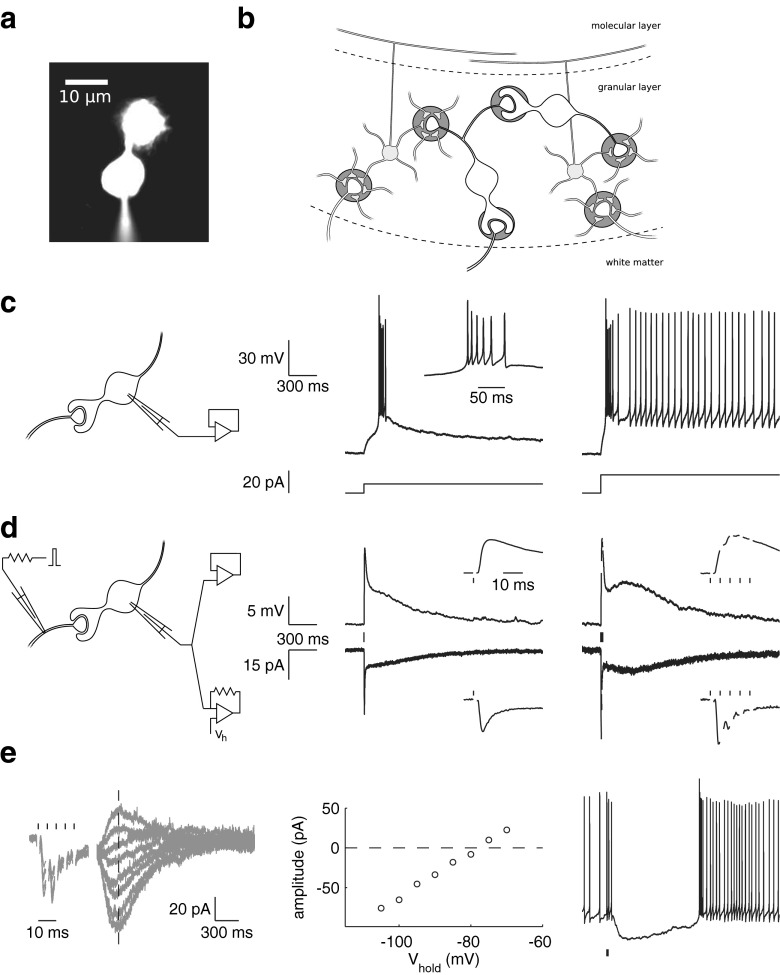
UBC characteristics. **a** A UBC filled with fluorescent dye through a patch pipette on the soma. **b** Connectivity in the vestibulocerebellar granular layer; inhibitory interneurons are not shown. Mossy fibers emanating from the white matter terminate in the granular layer, where they contact the dendrites of granule cells and UBCs within glomerular structures. UBC axons branch out within the granular layer, forming mossy fiber-like contacts with granule cells as well as other UBCs. **c** Typical UBC responses to current step injection in the current clamp mode. Responses were elicited from a −80-mV membrane potential. **d** Typical excitatory UBC responses to presynaptic stimulation in the current clamp (*top*) and voltage clamp (*bottom*) modes from the same cell (different cell as in **c**). Stimulus times are indicated by *vertical lines*. **e** Bimodal excitatory-inhibitory responses to presynaptic burst stimulation (five stimuli at 200 Hz). Fast excitatory EPSCs were followed by a slow inhibitory current with reversal potential around −80 mV. The *left panel* shows baseline-corrected voltage clamp recordings taken at holding potentials ranging from −105 to −70 mV. Slow-current amplitudes measured along the *dashed vertical line* are plotted in the *center panel* as a function of the holding potential. The slow current effectively inhibited action potential firing in the current clamp mode (*right panel*)

Forward processing, in particular the absence of recurrent excitation, is a defining feature of cerebellar architecture and computation. In the granular layer of the vestibulocerebellum, unipolar brush cells (UBCs) provide a powerful forward excitatory action onto granule cells through a cortex-intrinsic network of mossy fiber-like axons [1, 2]. UBCs are characterized by an elaborate brush-like dendrite (Fig. 1a) that forms an unusually extensive synaptic contact with a single presynaptic mossy fiber rosette [3]. This highly specialized configuration has been proposed to facilitate prolonged entrapment of glutamate in the synaptic cleft, underlying complex temporal transformations of incoming mossy fiber signals [4, 5]. In response to electrical stimulation of mossy fibers in vitro, UBCs exhibit protracted inward currents lasting hundreds of milliseconds [6, 7]. Persistent inward currents support periods of tonic action potential firing in UBCs, while phasic excitation can elicit strong action potential bursts and transiently increased firing rates [8, 9]. Such bimodal electroresponsiveness, coupled to the peculiar configuration of synaptic currents, could result in significant temporal transformation of incoming signals, while the strategic position in the granular layer circuitry allows a single UBC to directly affect hundreds of granule cells.

An estimated 50 % of mossy rosettes in the mouse nodulus originates from local UBC axons [1]. The postsynaptic targets have been identified at the ultrastructural level as granule cells, as well as other UBCs, as schematically depicted in Fig. 1b. UBC-granule cell synaptic connectivity is now well-established by electrophysiological recordings from postsynaptic granule cells in fresh cerebellar slices [2, 10]. Spontaneous synaptic events have been shown to occur in UBCs in organotypic slice cultures from 8-day-old mice [11], but such recordings have so far not been reported in fresh slices of more mature cerebellum. In this report, we show recordings of spontaneous and evoked (di)synaptic events in granule cells and UBCs in fresh cerebellar slices from juvenile mice (5–7 weeks). UBC-UBC connections displayed compound biphasic synaptic events that have also characterized responses to direct presynaptic stimulation of UBCs in previous studies. Such connections extend the cortex-intrinsic network of UBC axons pervading the granular layer circuitry, spreading a complex mixture of delayed and persistent signaling through chains of highly divergent mossy fiber projections [12, 13].

## Materials and Methods

The procedures for preparing and performing experiments were as described before [5]. Experiments were performed in the presence of 100 μM picrotoxin and 1 μM strychnine to block GABA-ergic and glycinergic inhibition. Unless otherwise stated, excitatory postsynaptic currents (EPSCs) were recorded at a holding potential of −80 mV. Coefficient of variation 2 (CV_2_) values were calculated as

$$ {\mathrm{CV}}_2=\frac{1}{N-1}\sum_{n=2}^N\frac{\left|{\mathrm{ISI}}_n-{{\mathrm{ISI}}_{n-}}_1\right|}{{\mathrm{ISI}}_n+{\mathrm{ISI}}_{n-1}}. $$

ISI_*n*_ is the *n*th of a total of *N* inter-spike intervals.

## Results

Whole-cell patch clamp recordings were obtained from granule cells and UBCs in lobule X and the ventral part of lobule IX in cerebellar slices of 5- to 7-week-old mice. In the voltage clamp mode, UBCs could be identified by their typical whole-cell capacitance and passive membrane resistance, while in current clamp, they were characterized by a bimodal response to stepwise somatic current injection [8]. An example of such a response is shown in Fig. 1c, where depolarizing current steps from a hyperpolarized membrane potential resulted in a burst of action potentials, followed by persistent regular action potential generation when current steps were sufficiently large. Another hallmark of UBC physiology is the prolonged NMDA- and/or AMPA-receptor-mediated inward current, lasting hundreds of milliseconds, observed after electrical stimulation of the presynaptic mossy fiber in vitro [6]. The AMPA-receptor-mediated component of this current is especially striking, as it develops a characteristic slow resurgent peak with increasing stimulation frequency [5], as exemplified in Fig. 1d. This peculiar behavior is believed to result from steady-state activation of AMPA receptors due to slow removal of glutamate from the synaptic cleft [4]. Metabotropic glutamate receptors mGluR1α and mGluR2 are heterogeneously expressed on extrasynaptic appendages in UBCs [14]. mGluR2 imparts inhibitory modulation on UBC activity through activation of G-protein-coupled inwardly rectifying potassium (GIRK) channels [15], which was recently shown to effectively inhibit action potential firing in response to high-frequency mossy fiber stimulation in a subset of UBCs [16]. An example of such an inhibitory current is shown in Fig. 1e, in which a burst of presynaptic stimuli induced a burst of conventional fast EPSCs, followed by a slow inhibitory current that reversed polarity around −80 mV.

**Fig. 2:**
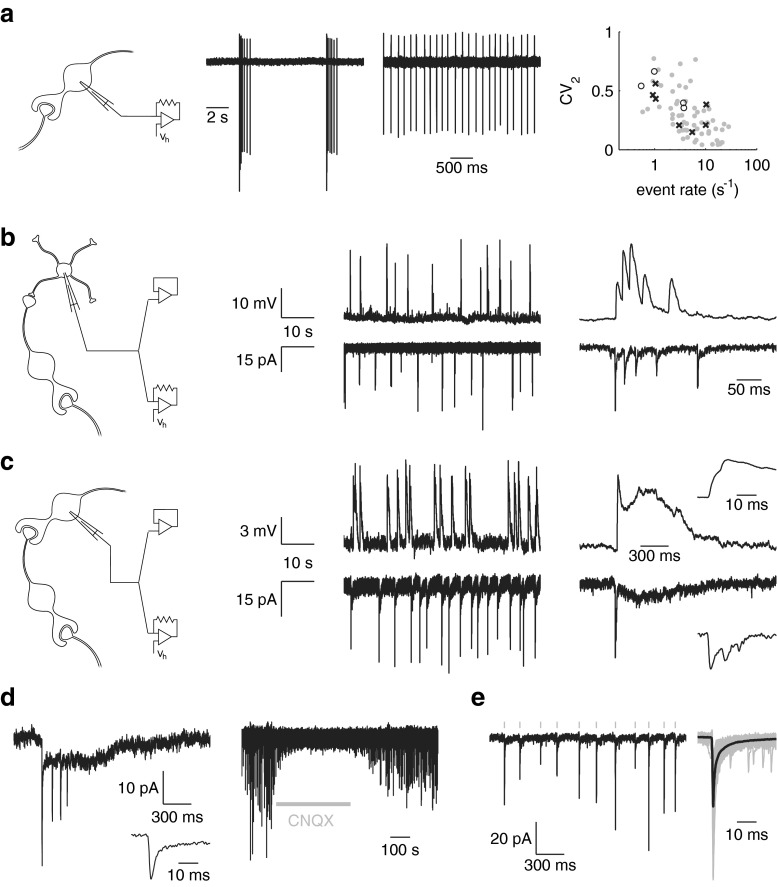
Spontaneous activity. **a** Examples of extracellular cell-attached recordings of spontaneous action potentials in two different UBCs. The very regular (low CV_2_) and very irregular (high CV_2_) examples spanned a range of activities recorded from 63 UBCs, shown as *gray dots in the rightmost panel*. Spontaneous synaptic events are also indicated, recorded from granule cells (*crosses*) and UBCs (*open circles*). **b** Spontaneous events in a granule cell recorded in the current clamp (*top*) and voltage clamp (*bottom*) modes. Events occurred in bursts, as shown in the examples enlarged in the *right panels*, putatively due to discharges of a presynaptic UBC. **c** Spontaneous synaptic events in a UBC, putatively due to discharges of a second presynaptic UBC. Bursts of fast events were followed by a slow resurgent tail, characteristic of UBC excitatory postsynaptic events. **d** Spontaneous burst of fast EPSCs in a UBC, followed by a slow current tail (different cell as in **c**). Bath application of AMPA receptor antagonist CNQX reversibly blocked synaptic events. **e** Example of regular EPSCs in a granule cell (different cell as in **b**). EPSCs detected in this cell were aligned (*gray*) and averaged (*black*), shown on the *right*

In fresh rat cerebellar slices, UBCs have been reported to be mostly inactive at rest [8, 7], while in mouse slices, a majority of UBCs were found to spontaneously generate regular action potential activity [17]. This difference has been attributed to the existence of two histochemically distinct classes of UBCs [9], although both classes of UBCs were found to be spontaneously active in slices of mouse dorsal cochlear nucleus [16]. Under our experimental conditions, 63 of 140 UBCs tested were spontaneously active during extracellular cell-attached recordings, displaying two main modes of activity: ongoing regular action potential generation or bursts of action potentials interrupting long periods of inactivity (Fig. 2a). Since external mossy fibers are not spontaneously active in slices, these patterns of spontaneous activity could serve as indicators for the presence of UBCs as presynaptic elements in the granular layer circuitry, manifest as spontaneous excitatory synaptic events in granule cells and UBCs. Figure 2b, c shows examples of whole-cell recordings from a granule cell and a UBC, respectively, displaying spontaneous events reminiscent of UBC action potential bursts. Despite the apparently similar presynaptic activity, the two cell types experienced very different postsynaptic effects. Conventional fast synaptic events in the UBC were followed by a slow resurgent tail, shaping the protracted compound event that characterizes UBC excitatory synaptic transmission. Another example of such an event recorded from another UBC is shown in Fig. 2d. These events were reversibly suppressed by bath application of AMPA receptor antagonist CNQX. In Fig. 2e, an example recording is shown from a granule cell, displaying spontaneous events resembling the regular mode of UBC activity.

**Fig. 3:**
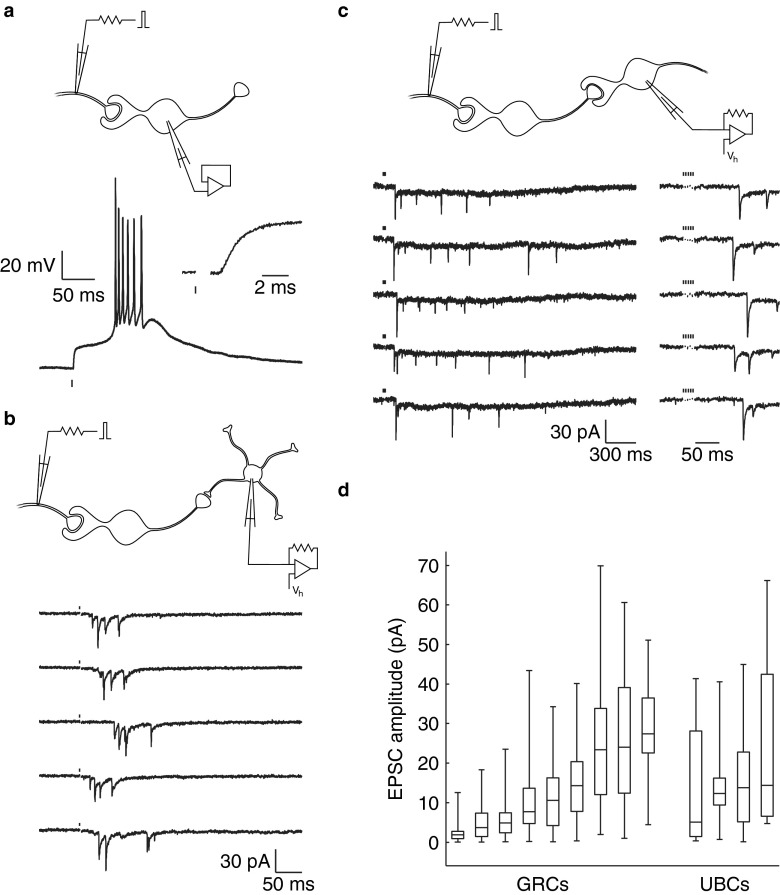
Evoked responses. **a** Burst response of a UBC in the current clamp mode due to a presynaptic electrical stimulus. The short delay to EPSP onset (*inset*) indicated a monosynaptic connection. Stimulation artifacts were removed for clarity. **b** Bursts of EPSCs in a granule cell in response to presynaptic stimulation, with an average first-peak delay of 35 ms. The responses are reminiscent of the evoked UBC burst shown in panel **a. c** Trains of EPSCs in a UBC in response to presynaptic stimulation (five stimuli at 200 Hz), with an average first-peak delay of 97 ms. The slow EPSC tails fused into a current plateau lasting several seconds. **d** Amplitudes of spontaneous and evoked disynaptic EPSCs due to a presynaptic UBC, recorded from nine granule cells (GRCs) and four UBCs. *Boxes* indicate the first, second (median), and third quartiles. *Whiskers* indicate the lowest and highest occurring values

UBC EPSPs can couple to intrinsic membrane mechanisms to produce a burst of action potentials in response to presynaptic stimulation (Fig. 3a). Such evoked UBC action potential bursts are likely to have caused the bursts of EPSCs observed in the granule cell in Fig. 3b, in response to electrical stimulation of the white matter. The average delay of the first EPSC in the burst was 35 ms, indicating disynaptic transmission via a presynaptic UBC. In an analogous experiment, trains of EPSCs were recorded from a postsynaptic UBC with an average onset delay of 97 ms (Fig. 3c), indicating delayed activation of long-lasting depolarization in a presynaptic UBC [7]. As was previously observed in response to direct monosynaptic stimulation [5], fast EPSCs underwent strong depression during the trains, and integration of the slow EPSC tails supported a current plateau lasting seconds. An overview of fast EPSC amplitudes recorded from granule cells and UBCs is shown in Fig. 3d.

## Discussion

Accurate timing of neural commands is central to cerebellar function, and accordingly, several complementary mechanisms have been identified that tightly control granule cell activity with (sub-)millisecond precision [18]. The presence of UBCs in the granular layer circuitry might facilitate coordination of activity on longer time scales. In addition to contacting granule cells, UBCs have been shown at the ultrastructural level to synapse on other UBCs as well [2], and spontaneous synaptic events have been observed in UBCs in organotypic cultures of the mouse nodulus [11]. Here, we show that chains of interconnected UBCs functioned in fresh slices of the juvenile mouse cerebellum and that UBC-UBC connections displayed protracted biphasic synaptic events. Such chains of UBCs could serve to broaden the temporal window of delayed activation spreading through the granular layer [13].

UBC synaptic excitation is balanced by GABA- and glycine-mediated synaptic inhibition [19] and further complemented by modulation through extrasynaptic receptors [7, 16]. Although we did not find evidence for slow inhibitory currents following spontaneous presynaptic bursts in UBCs, the localization of mGluRs on extrasynaptic appendages within the glial ensheathment of the glomerular UBC synapse suggests they may predominantly sense locally spilled glutamate [14]. It remains to be determined how the various fast and slow modulatory signals cooperate to coordinate UBC activity.

Estimates from organotypic cultures indicate that ~50 % of mossy rosettes in the nodulus originate from UBCs and that ~70 % of these rosettes are involved in UBC-UBC contacts [1]. While almost half of our UBCs displayed spontaneous activity at rest, we found that spontaneous synaptic events occurred in only a very small fraction of UBCs tested (4 of 140). This discrepancy is most likely due to our experimental preparation. For whole-cell recordings, we were limited to studying relatively superficial UBCs, with corresponding presynaptic axons that are at high risk of being cut during the slicing procedure, especially since they are known to often traverse large portions of the lobule.

UBCs seem specialized to recode or emphasize a particular type of information encoded in (a particular subset of) external mossy fibers. How UBCs could communicate this information to downstream neurons in vivo depends on their physiological state and the nature of the input signal. From a hyperpolarized membrane potential, UBCs are able to generate bursts of action potentials, which appear well-suited to accurately relay temporal information with high fidelity. On the other hand, many UBCs display tonic regular activity at rest both in vitro and in vivo and might in such cases communicate through delayed and persistent changes in their ongoing tonic activity [10, 20]. As differentiation of neuronal phenotypes may be regulated by presynaptic partners, phasic and tonic UBC subtypes may be connected in chains according to a general organizing principle, perhaps in such a way as to increase the range of attainable signal delays while maintaining temporal segregation of inputs [9].
